# Efficacy and safety of selective JAK 1 inhibitor filgotinib in active rheumatoid arthritis patients with inadequate response to methotrexate: comparative study with filgotinib and tocilizumab examined by clinical index as well as musculoskeletal ultrasound assessment (TRANSFORM study): study protocol for a randomized, open-label, parallel-group, multicenter, and non-inferiority clinical trial

**DOI:** 10.1186/s13063-023-07176-5

**Published:** 2023-03-03

**Authors:** Toshimasa Shimizu, Shin-ya Kawashiri, Shimpei Morimoto, Yurika Kawazoe, Shohei Kuroda, Rina Kawasaki, Yasuko Ito, Rieko Kiya, Shuntaro Sato, Hiroshi Yamamoto, Atsushi Kawakami

**Affiliations:** 1 Clinical Research Center, Nagasaki University Hospital, Nagasaki, Japan; 2 Department of Immunology and Rheumatology, Division of Advanced Preventive Medical Sciences, Nagasaki University Graduate School of Biomedical Sciences, Nagasaki, Japan; 3 Department of Community Medicine, Division of Advanced Preventive Medical Sciences, Nagasaki University Graduate School of Biomedical Sciences, 1-12-4 Sakamoto, Nagasaki, 852-8523 Japan; 4 Innovation Platform & Office for Precision Medicine, Nagasaki University Graduate School of Biomedical Sciences, Nagasaki, Japan

**Keywords:** Rheumatoid arthritis, Filgotinib, JAK inhibitor, Tocilizumab, IL-6 inhibitor, Musculoskeletal ultrasound, Biomarker

## Abstract

**Background:**

Administration of Janus kinase (JAK) inhibitors and biological disease-modifying antirheumatic drugs has dramatically improved even the clinical outcomes in patients with rheumatoid arthritis (RA) and an inadequate response to methotrexate (MTX). Dysregulation of JAK-STAT pathways via overproduction of cytokines, such as interleukin-6, is involved in the pathogenesis of RA. Filgotinib is a selective JAK1 inhibitor pending approval for use in RA. By inhibition of the JAK-STAT pathway, filgotinib is effective in suppressing disease activity and preventing the progression of joint destruction. Similarly, interleukin-6 inhibitors such as tocilizumab also inhibit the JAK-STAT pathways by inhibition of interleukin-6 signaling. We present the protocol for a study that will evaluate whether the effectiveness of filgotinib monotherapy is non-inferior to that of tocilizumab monotherapy in RA patients with an inadequate response to MTX.

**Methods:**

This study is an interventional, multicenter, randomized, open-label, parallel-group, and non-inferiority clinical trial with a 52-week follow-up. Study participants will be 400 RA patients with at least moderate disease activity during treatment with MTX. Participants will be randomized in a 1:1 ratio to administer filgotinib monotherapy or subcutaneous tocilizumab monotherapy switched from MTX. We will evaluate disease activity by measuring clinical disease activity indices and by using musculoskeletal ultrasound (MSUS). The primary endpoint is the proportion of patients who achieve an American College of Rheumatology 50 response at week 12. Secondary endpoints are changes from baseline in the MSUS scores. We will also comprehensively analyze serum levels of multiple biomarkers, such as cytokines and chemokines.

**Discussion:**

The study results are expected to show the non-inferiority of the effectiveness of filgotinib monotherapy to that of tocilizumab monotherapy in RA patients with inadequate response to MTX. The strength of this study is its prospective evaluation of therapeutic efficacy using not only clinical disease activity indices, but also MSUS, which accurately and objectively evaluates disease activity at the joint level among patients drawn from multiple centers with a standardized evaluation by MSUS. We will evaluate the effectiveness of both drugs by integrating multilateral assessments—clinical disease activity indices, MSUS findings, and serum biomarkers.

**Trial registration:**

Japan Registry of Clinical Trials (https://jrct.niph.go.jp) jRCTs071200107. Registered on March 3, 2021. ClinicalTrials.gov NCT05090410. Registered on October 22, 2021.

**Supplementary Information:**

The online version contains supplementary material available at 10.1186/s13063-023-07176-5.

## Background

Rheumatoid arthritis (RA) is a chronic, systemic inflammatory disease that primarily involves the synovial joints [1]. Uncontrolled disease activity of RA may lead to joint destruction and deformity, causing impaired quality of life. Therefore, tight control of disease activity using the treat-to-target strategy is recommended to prevent joint destruction [2].

The treatment gold standard is conventional synthetic disease-modifying antirheumatic drugs (csDMARDs) with methotrexate (MTX) as the first-line agent in patients with active RA; however, a considerable proportion of the patients are refractory to treatment with MTX. Furthermore, a continuation of MTX is limited by adverse events and poor tolerability. Based on the European League Against Rheumatism (EULAR) recommendations, the choice of DMARDs in the second phase of treatment is important for patients with inadequate or intolerant to MTX [3]. Biological DMARDs (bDMARDs), which are mainly used in the second phase after inadequate response to MTX, have provided better clinical outcomes, including the achievement of clinical remission for patients with RA. In recent years, Janus kinase (JAK) inhibitors have emerged as the second choice of treatment for RA patients with an inadequate response to MTX [3].

Overproduction and overexpression of proinflammatory cytokines, such as interleukin-6 (IL-6), bind to its receptors to activate the JAK-signal transducer and activator of transcription (STAT) signaling pathways, which are involved in the pathogenesis of RA [4]. Thus, JAK inhibitors are effective in suppressing RA disease activity by inhibition of the JAK-STAT signaling pathways.

Filgotinib is a preferential JAK1 inhibitor that was developed by Gilead (Foster City, CA, USA). In previous studies, almost 50% of RA patients for whom filgotinib was added achieved clinical remission after inadequate response to csDMARDs, including MTX [5, 6]. In addition, the effects of JAK inhibitors including filgotinib are non-inferior or superior to those of tumor necrosis factor (TNF) inhibitors in patients with active RA and an inadequate response to MTX [6–9]; however, to date, no head-to-head comparison between JAK inhibitors and IL-6 inhibitors has been performed. As noted previously, JAK inhibitors inhibit signal transduction of JAK-STAT pathways, whereas IL-6 inhibitors such as tocilizumab also inhibit JAK-STAT pathways by inhibition of IL-6 signaling [4]. Therefore, it is important to investigate whether the effectiveness of JAK inhibitors is non-inferior to that of IL-6 inhibitors in active RA patients with an inadequate response to MTX.

Musculoskeletal ultrasound (MSUS) has become widely used for the evaluation of disease activity in RA [10, 11]. According to experts in MSUS, RA patients treated with DMARDs should undergo MSUS because this assessment better shows synovial inflammation compared with a clinical examination [10, 11]; they also indicated that MSUS to assess therapeutic response can be of great benefit in clinical practice [10–13]. As a noninvasive, objective, relatively inexpensive, and repeatable imaging modality, MSUS is suitable for treatment monitoring [10, 11].

As noted previously, clinical remission can be achieved in a relatively large number of RA patients by introducing JAK inhibitors or bDMARDs; however, even in patients who achieve clinical remission, residual synovitis may be detected on MSUS [14, 15]. Residual synovitis is an important finding that predicts joint destruction and clinical relapse [16, 17]. Thus, it is important to accurately evaluate disease activity at the joint level by using MSUS as well as clinical disease activity indices, including subjective parameters. In this study, we will use MSUS assessments to determine whether filgotinib monotherapy is non-inferior to tocilizumab monotherapy in RA patients with inadequate response to MTX. This research is critical because a multicenter collaborative study that prospectively evaluates disease activity using MSUS standardized at a high level is rare, even worldwide. We will also evaluate changes in disease activity using MSUS and clinical disease activity indices to more accurately assess disease activity in this population. In addition, we will comprehensively analyze serum levels of many biomarkers, such as cytokines and chemokines.

We named this clinical trial “Efficacy and safety of selective JAK1 inhibitor Filgotinib in active rheumatoid arthritis patients with inadequate response to methotrexate: Comparative study with Filgotinib and Tocilizumab examined by clinical index as well as musculoskeletal ultrasound assessment (TRANSFORM study).” Herein we describe the final study protocol (version 1.4; October 27, 2021).

## Objectives

### Primary objective

The primary objective of the study is to evaluate whether the effects of filgotinib monotherapy are non-inferior to those of tocilizumab monotherapy in RA patients with inadequate response to MTX.

### Secondary objectives

The secondary objectives of the study are to evaluate changes in patient parameters, including clinical disease activity indices, MSUS scores, serum biomarkers, patient-reported outcomes, and van der Heijde-modified total Sharp score (mTSS) after administration of filgotinib or tocilizumab.

## Methods/design

### Study design

**Fig. 1:**
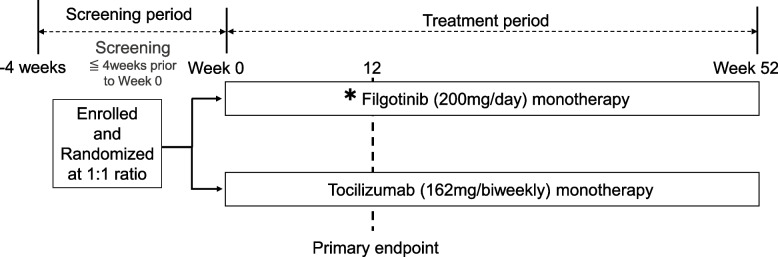
Study design. The asterisk indicates that patients with moderate renal dysfunction (estimated glomerular filtration rate 30–60 mL/min/1.73 m^2^) will be allowed to be administered filgotinib 100 mg/day

The study design is in accordance with the Standard Protocol Items: Recommendations for Interventional Trials and Consolidated Standards of Reporting Trials 2010 guidelines [18, 19] (Additional file 1). The study is a prospective, randomized, open-label, two-arm, and interventional clinical trial. It will be conducted at the following 55 centers: Nagasaki University Hospital, Hokkaido University Hospital, University of Tsukuba Hospital, Saitama Medical University Hospital, Chiba University Hospital, Yokohama City University Medical Center, Yokohama City University Hospital, Yokohama Minami Kyosai Hospital, Yoshimi Hospital, Chubu-Rosai Hospital, Kyoto University Hospital, University Hospital Kyoto Prefectural University of Medicine, Osaka Medical and Pharmaceutical University Hospital, Osaka City University Hospital, National Hospital Organization Osaka Minami Medical Center, Kindai University School of Hospital, Kita-Harima Medical Center, Kobe University Hospital, Yamaguchi Prefectural Welfare Agricultural Cooperative Association Nagato General Hospital, Kagawa University Hospital, Ehime University Hospital, Kochi Medical School Hospital, Hospital of the University of Occupational and Environmental Health, Tobata General Hospital, PS Clinic, National Hospital Organization Ureshino Medical Center, Miyakonojyo Medical Center, Miyazaki Zenjinkai Hospital, Yoshitama Clinic for Rheumatic Diseases, Japanese Red Cross Nagasaki Genbaku Hospital, National Hospital Organization Nagasaki Medical Center, Sasebo Chuo Hospital, Fukushima Medical University Hospital, Toho University Ohashi Medical Center, Niigata Rheumatic Center, Nara Medical University Hospital, Tohoku Medical and Pharmaceutical University Wakabayashi Hospital, Aomori Prefectural Central Hospital, Nippon Medical School Hospital, St. Marianna Medical University Hospital, Tohoku University Hospital, Juntendo University Hospital, Showa University East Hospital, University of Yamanashi Hospital, Kumamoto Shinto General Hospital, National Hospital Organization Chiba-East-Hospital, Keio University Hospital, University of Tokyo Hospital, Yukawa Rheumatology Clinic, Utazu Hospital, Saga University Hospital, Niigata University Medical and Dental Hospital, Nagoya Rheumatology Clinic, National Center for Geriatrics and Gerontology, and Matsuyama Red Cross Hospital. In total, 400 patients with RA will be assigned to switch from MTX ± other csDMARDs to filgotinib or tocilizumab. The duration of the intervention will be 52 weeks. The study design is summarized in Fig. 1.

### Approvals

The study was approved by the certified review board of Nagasaki University (approval no. CRB20-026). The study was registered in the Japan Registry of Clinical Trials (https://jrct.niph.go.jp) as jRCTs071200107 and in ClinicalTrials.gov on October 22, 2021, as NCT05090410. We will conduct the study in accordance with the principles of the Declaration of Helsinki and Clinical Trials Act (Act No. 16 of April 14, 2017), the Act on the Protection of Personal Information and related regulatory notifications, and this clinical study protocol. Potential participants will be provided with an explanation of the study by their treating rheumatologist and will be asked to voluntarily sign an informed consent form before participation. This consent will also include the use of the data for future research.

Any modifications of the protocol must be approved by the certified review board of Nagasaki University. In addition, the sponsor will report to the investigator based on the results of the review and will obtain approval from the administrators of the participating medical institutions.

### Participants

#### Inclusion criteria

Patients must meet all of the following requirements to be considered for study enrollment: (1) age ≥ 20 years; (2) diagnosis of RA based on the American College of Rheumatology (ACR)/EULAR 2010 RA Classification Criteria [20]; (3) at least moderate disease activity, defined as a Disease Activity Score-28 (DAS28)-erythrocyte sedimentation rate (ESR) ≥ 3.2 at the eligibility evaluation; (4) MTX treatment for ≥ 8 weeks before providing consent, including ≥ 4 weeks at the same doses of 8–16 mg/week (stable doses of < 8 mg/week are allowed only in the presence of intolerance to higher doses); and (5) ability and willingness to provide written informed consent and comply with the study protocol requirements.

#### Exclusion criteria

The exclusion criteria are as follows: (1) concurrent use of a corticosteroid equivalent to > 5 mg/day of prednisolone; (2) a contraindication for filgotinib or tocilizumab; (3) previous use of a JAK inhibitor or IL-6 inhibitor; (4) treatment with a corticosteroid and csDMARD and change of dose within 4 weeks before providing consent; (5) treatment with a biologic DMARD or a biosimilar DMARD (i.e., infliximab, biosimilar of infliximab, adalimumab, biosimilar of adalimumab, golimumab, certolizumab pegol, or abatacept) within 8 weeks before providing consent; (6) treatment with a TNF inhibitor (i.e., etanercept or biosimilar of etanercept) within 4 weeks before providing consent; (7) use of a prohibited drug or therapy, other than the agents listed, within 4 weeks before providing consent; (8) complication causing musculoskeletal disorders other than RA (i.e., ankylosing spondyloarthritis, reactive arthritis, psoriatic arthritis, crystal-induced arthritis, systemic lupus erythematosus, systemic scleroderma, inflammatory myopathy, or mixed connective tissue disease); (9) current pregnancy, breastfeeding, or nonadherence with a medically approved contraceptive regimen during and 12 months after the study period; or (10) inappropriateness for study inclusion as determined by the investigator.

#### Intervention

Patients will be randomized in a 1:1 ratio to the administration of filgotinib 200 mg/day or subcutaneous tocilizumab 162 mg/biweekly switched from MTX ± other csDMARDs throughout the study period. Patients with moderate renal dysfunction (estimated glomerular filtration rate 30–60 mL/min/1.73 m^2^) will be allowed to be administered filgotinib 100 mg/day.

All patients must continue to receive the same doses of corticosteroid that they were receiving before providing consent throughout the study period. During the study period, the following treatments are prohibited: administration of a bDMARD, except tocilizumab, or JAK inhibitor, except for filgotinib; concomitant use of an immunosuppressant (azathioprine, cyclophosphamide, cyclosporine), csDMARD, or oral corticosteroids equivalent to more than 5 mg/day of prednisolone, in addition to intra-articular corticosteroid injections at joints, and nonsteroidal anti-inflammatory drug suppositories. During the study period, the dose of any oral nonsteroidal anti-inflammatory drug can be modified within the range of its approved doses in Japan.

#### Patient discontinuation criteria

A patient may be prematurely withdrawn from the study for the following reasons:

- Filgotinib must be discontinued for > 7 consecutive days in the filgotinib group.
- Tocilizumab must be discontinued for ≥ 2 consecutive injections in the tocilizumab group.
- The patient asks to leave the trial.
- The patient asks to change or discontinue the treatment.
- Continuing participation is inadvisable due to adverse event(s).
- The patient becomes pregnant.
- At the principal investigator’s discretion, the continuation of the trial would be detrimental to the patient’s well-being.

The patient with discontinuation will receive the outcome measurement at the time of discontinuation (Table 1), if the patient’s cooperation has been obtained from the patient.

**Table 1:**
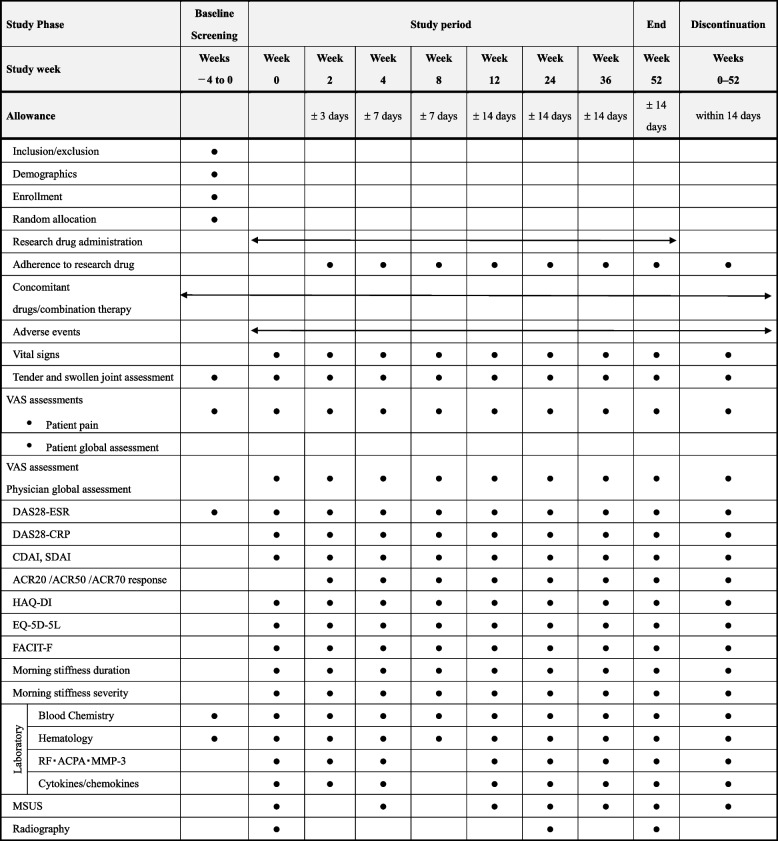
Treatment schedule and outcome measures

*ACPA* anti-cyclic citrullinated peptide antibody, *ACR* American College of Rheumatology, *CDAI* clinical disease activity index. *CRP* C-reactive protein, *DAS28* Disease Activity Score-28, *EQ-5D-5L* EuroQol-5 Dimension 5-Level, *ESR* erythrocyte sedimentation rate, *FACIT-F* Functional Assessment of Chronic Illness-Fatigue, *HAQ-DI* Health Assessment Questionnaire Disability Index, *MMP-3* matrix metalloproteinase-3, *MSUS* musculoskeletal ultrasound, *RF* rheumatoid factor, *SDAI* simplified disease activity index, *VAS* visual analog scale

### Outcome measurements

**Fig. 2:**
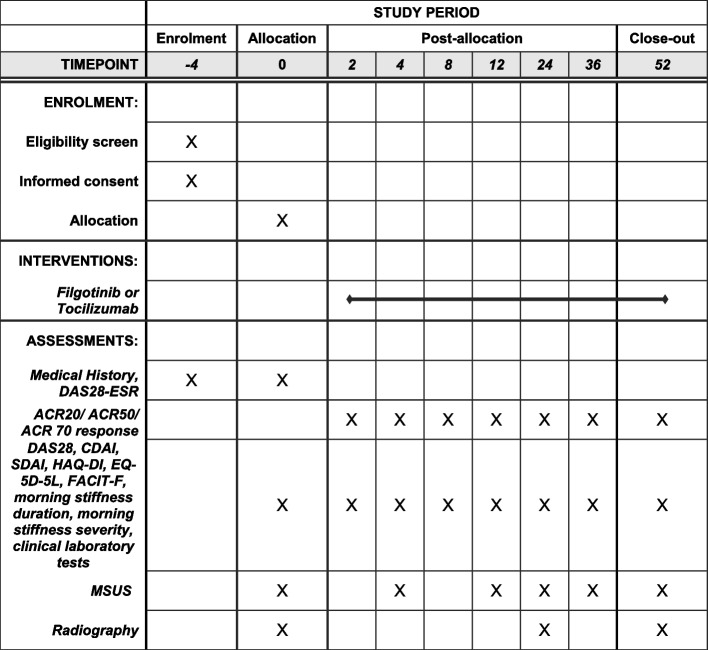
The schedule of enrolment, interventions, and assessments. ACR, American College of Rheumatology; CDAI, clinical disease activity index; DAS28, Disease Activity Score-28; EQ-5D-5L, EuroQol-5 Dimension 5-Level; ESR, erythrocyte sedimentation rate; FACIT-F, Functional Assessment of Chronic Illness-Fatigue; HAQ-DI, Health Assessment Questionnaire Disability Index; MSUS, musculoskeletal ultrasound; SDAI, simplified disease activity index

Study visits will be conducted at baseline and 2, 4, 8, 12, 24, 36, and 52 weeks after the administration of filgotinib or tocilizumab. The assessments are presented in Table 1 and Fig. 2. Clinical physicians will be blinded to the results of the joint assessments by MSUS.

#### Clinical disease activity

Clinical disease activity was evaluated by each attending physician (Japan College of Rheumatology [JCR]-certified rheumatologists) based on the values of the ACR core set, the Disease Activity Score-28 (DAS28)-ESR and DAS28-C-reactive protein (CRP), and the clinical disease activity index (CDAI) and simplified disease activity index (SDAI) level. For tender joints (68 joints) and swollen joints (66 joints), improvement on 3 of the following 5 assessments will define the ACR response: (1) patient’s global assessment, (2) patient’s pain assessment, (3) evaluator’s global assessment, (4) Health Assessment Questionnaire Disability Index [HAQ-DI], and (5) CRP or ESR. The rates of the ACR response are defined as *ACR20 response*, *ACR50 response*, and *ACR70 response* based on an improvement of ≥ 20%, ≥ 50%, or ≥ 70%, respectively. Each patient’s global and pain assessment and the evaluator’s global assessment will be established on a 0–100-mm visual analog scale. The patients’ reported outcome will be evaluated by the morning stiffness duration and severity, EuroQol 5 Dimensions 5-Level (EQ-5D-5L), and Functional Assessment of Chronic Illness-Fatigue (FACIT-F).

#### MSUS assessments

Participants will undergo imaging by MSUS at baseline and 4, 12, 24, 36, and 52 weeks performed by one of the JCR-certified sonographers. A systematic multiplanar grayscale (GS) and power Doppler (PD) examination of each patient’s joints will be performed using a multifrequency linear transducer (12–24 MHz). Depending on which Doppler modality is the most sensitive on the individual machines, PD will be used. The Doppler settings will be adjusted at each hospital according to published recommendations [21]. During the study, no change in MSUS settings and no software upgrading will occur.

Articular synovitis will be assessed by MSUS on dorsal views of 22 joints: bilateral wrist joints, 1st–5th metacarpophalangeal joints, interphalangeal joints, and 2nd–5th proximal interphalangeal joints. GS grade semiquantitatively scores the degree of synovial hypertrophy of each joint as within normal limits (grade 0), minimal (grade 1), moderate (grade 2), or severe (grade 3). In addition, PD grade semiquantitatively scores the degree of synovial PD signal of each joint as within the normal range (grade 0), minimal (grade 1), moderate (grade 2), and severe (grade 3) [22, 23]. The sum of the GS or PD scores is considered to be the total GS or PD scores, respectively. We will also assess the Outcome Measures in Rheumatology (OMERACT)-EULAR combined PDUS score (i.e., the combined PD score) and Global OMERACT-EULAR Synovitis Score [22, 23]. The combined PD score is combined with synovial hypertrophy shown by GS and PD [22, 23].

#### Radiographic imaging

Radiographic imaging of the bilateral hands (posteroanterior view) and feet (anteroposterior view) will be conducted. Joint damage progression will be evaluated based on the vdH-mTSS method, including 16 areas in each hand for erosion and 15 for joint-space narrowing [24].

#### Biomarker measurements

Serum concentrations of the following biomarkers will be measured. Rheumatoid factor (RF) will be measured using latex agglutination turbidimetric immunoassay (LZ test “Eiken” RF). Anti-cyclic citrullinated peptide antibodies will be measured using a chemiluminescent immunoassay (STACIA MEBLux test CCP). Matrix metalloproteinase-3 (MMP-3) was measured using a latex turbidimetric immunoassay (Panaclear MMP-3 “Latex”). Multiplex cytokine/chemokine bead assays will be performed using diluted serum supernatants and MILLIPLEX MAP Human Cytokine/Chemokine Magnetic Bead Panel (Merck Millipore)–Bio-Plex Pro Human Cytokine Assays (Bio-Rad) analyzed with a Bio-Plex MAGPIX Multiplex Reader (Bio-Rad), according to the manufacturer’s instructions.

The cytokines/chemokines that are measured by the bead panel include interleukin (IL)-1α, IL-1β, IL-1 receptor antagonist, IL-2, IL-4, IL-5, IL-6, IL-7, IL-8, IL-10, IL-12 (p40), IL-12 (p70), IL-13, IL-15, IL-17A, IL-17F, IL-18, IL-22, IL-27, interferon-gamma (IFN-γ), IFN-α2, CXCL1 (growth-related oncogene), granulocyte–macrophage colony-stimulating factor, granulocyte colony-stimulating factor, CX3CL1 (fractalkine), flt-3 ligand, fibroblast growth factor-2, eotaxin, epidermal growth factor, vascular endothelial growth factor, platelet-derived growth factor-AA, soluble CD40 ligand, TNF-α, TNF-β, transforming growth factor-α, CCL4 (macrophage inflammatory protein [MIP]-1β), CCL3 (MIP-1α), CCL22 (macrophage-derived chemokine), CCL7 (monocyte chemotactic protein-3), CCL2 (monocyte chemotactic protein-1), CXCL10 (IFN-γ-inducible protein-10), vascular cell adhesion molecule-1, and intercellular adhesion molecule-1. The serum IL-6 and TNF-α levels will be measured using specific enzyme-linked immunosorbent assay kits (R&D Systems).

Residual serum samples will be stored at Nagasaki University Hospital for 5 years after the completion of the study for future research.

### Study endpoints

#### Primary endpoint

The primary endpoint is the ACR50 response at week 12.

#### Secondary endpoints

The secondary endpoints of this study are as follows: (1) ACR50 response at weeks 2, 4, 8, 24, 36, and 52; (2) ACR20 response at weeks 2, 4, 8, 12, 24, 36, and 52; (3) ACR70 response at weeks 2, 4, 8, 12, 24, 36, and 52; (4) changes in the CDAI and SDAI values from baseline to weeks 2, 4, 8, 12, 24, 36, and 52; (5) changes in the DAS28-ESR and DAS28-CRP values from baseline to weeks 2, 4, 8, 12, 24, 36, and 52; (6) changes in the serum levels of biomarkers from baseline to weeks 2, 4, 12, 24, 36, and 52; (7) changes in the total PD and GS scores and combined PD score from baseline to weeks 4, 12, 24, 36, and 52; (8) change in vdH-mTSS from baseline to weeks 24 and 52; (9) change in the HAQ-DI data from baseline to weeks 2, 4, 8, 12, 24, 36, and 52; (10) change in the EQ-5D-5L data from baseline to weeks 2, 4, 8, 12, 24, 36, and 52; (11) change in the FACIT-F data from baseline to weeks 2, 4, 8, 12, 24, 36, and 52; and (12) changes in the morning stiffness duration and morning stiffness activity from baseline to weeks 2, 4, 8, 12, 24, 36, and 52.

### Adverse events

All adverse events that occur between the baseline visit and the end of week 52 will be recorded in the medical records. If necessary, the investigators will administer treatment. In addition, all adverse events will be evaluated for severity, predictability, causality to the study, seriousness, and outcome. A *serious adverse event* is defined as any adverse reaction resulting in any of the following outcomes: a life-threatening condition or death; a condition that requires inpatient hospitalization or prolongation of existing hospitalization; and a condition threatening to cause disability or disability, congenital anomaly, or congenital anomaly. All serious adverse events will be documented in the medical records and reported to the certified review board by the responsible investigator in accordance with Japanese regulations.

### Data collection, management, and monitoring

The patient data in an online, web-based electronic data capture (EDC) system will be regarded as an electronic case report form. The biomarker data (e.g., cytokines), MSUS reassessment results, and vdH-mTSS results are collected as external data in an electronic format (Excel or CSV format), instead of entering in the EDC. According to the Table 1 schedule, the investigator will collect data at each patient visit during the study. Appropriate and authorized persons (investigators, clinical trial physicians, and clinical trial collaborators) will be provided access to the EDC system and will be able to enter and modify collected patient data into an EDC system. All data recorded in the electronic case report form must be consistent with the original materials.

All study findings and documents will be regarded as confidential. Each patient will be identified on the electronic case report form by an anonymous number, not by name. To ensure confidentiality, the investigator will maintain the anonymity of documents that identify the patient. During the study, I’cros Co., Ltd. (Fukuoka, Japan), a site management organization (SMO) that is independent of the sponsor/investigators and has no competing interest, will perform regular site visits to review protocol compliance, conduct source data verification, assess drug accountability and management, and ensure that the study is being conducted according to relevant regulatory and protocol requirements. After the monitoring has been conducted, the monitor will report the results of the monitoring to the sponsor/investigators. The sponsor will manage and supervise the monitoring of each site to ensure ethics, safety, and data reliability.

### Randomization

The sponsor/investigators will use the EDC system (DATATRAK) built by an independent data manager from the sponsor/investigators or founders to randomly assign patients to receive filgotinib alone or tocilizumab subcutaneously in a 1:1 ratio using computer-generated random numbers automatically. Our randomization method is the minimization method and stratified factors are disease duration of RA (< 2 years and ≥ 2 years), disease activity (DAS28-ESR > 5.1 and ≤ 5.1), weight (< 60 kg and ≥ 60 kg), and dose of MTX at randomization (< 10 mg/week and ≥ 10 mg/week).

### Sample size

Based on the following description of power analysis, the total required number of participants for randomization was estimated to be 400. First, we estimated the sample size to obtain a statistical power of 0.80 in the primary analysis using 2000 pairs of binary sequences generated from the Bernoulli process with the parameter *p* = 0.40 and the length of a predetermined value *n*. The value of *p*, 0.40, was decided based on a previous report [25]. Our null hypothesis is that the difference in the proportions between the 2 groups is higher than the non-inferiority margin, which is 0.15, in our primary analysis. We determined the minimum of the *n* with which the probability of > 0.80 in rejection of the null hypothesis by counting the number of pairs of binary sequences with which the upper limit of the 2-sided 95% confidence intervals of proportions’ difference [26] < 0.15 among 2000 pairs of binary sequences. Consequently, the minimum of *n*, which corresponded to the sample size to obtain a statistical power over 0.80, was estimated to be 176 participants per group. Then, we set the target number of patients to 200 participants per group, assuming that 12% of enrolled participants would be excluded from the per-protocol set. The power analysis was conducted in the R: a language and environment for statistical computing [27]. The Wilson score interval of difference was calculated via the diffscoreci (score interval for the difference of proportions) function in the PropCIs package version 0.3.0.

### Statistical analysis method

Only for the primary analyses on the primary endpoint will we state the test size, whereas *p* values obtained from other analyses will be reported as descriptive statistics [28]. The *full analysis set* consists of the participants with the randomization results and the baseline measurement results of the ACR core set components. Each participant will be analyzed as a member of a treatment group into which the participant was allocated at randomization, regardless of the treatment during the study observation period. The *per-protocol set* consists of a subset of the full analysis set and includes the participants with week 12 results for the ACR core set components. The other inclusion criteria for the per-protocol set can be added before the database lock.

The primary analysis is a hypothesis test for the non-inferiority in the per-protocol set. The difference in the primary endpoint between the treatment groups will be estimated as the 2-sided 95% confidence interval of the difference in the proportions. The non-inferiority margin is set at 0.15. The confidence interval for differences between the 2 groups will be calculated from the rate difference in stratified pairs of proportions with the allocation factors [29]. The non-inferiority margin was decided by discussions within the research group that consisted of the physicians, including the principal investigator of this study.

If we observe the non-inferiority in the primary analysis, we will then proceed to a hypothesis test for the superiority in the per-protocol set. The null hypothesis is that the odds ratio of the treatment group to the primary endpoint is 1. The null hypothesis will be tested by the Fisher exact test of the 2-tailed test size of 0.05.

The nested multiple imputations will be performed for missing measurements at the primary endpoint. As a sensitivity analysis of the assumptions in the applied imputation methods, the primary analyses will be reperformed using the following imputation rule for the missing data instead of the nested multiple imputations: the result that “ACR50 response was not achieved” will be imputed for the missing data in the filgotinib group, whereas “ACR50 response was achieved” will be imputed for the missing data in the tocilizumab group.

## Discussion

The main purpose of this clinical trial was to evaluate whether filgotinib monotherapy is non-inferior to tocilizumab monotherapy in the improvement of disease activity in RA patients with inadequate response to MTX. The introduction of JAK inhibitors and bDMARDs into clinical practice has dramatically improved the management of a number of immune-mediated inflammatory diseases, including RA [3]. Several studies have shown the effectiveness of JAK inhibitors compared with bDMARDs in patients with RA [6–9, 30]. With regard to the effectiveness of filgotinib, the FINCH 1 trial showed that filgotinib 200 mg/day + MTX was non-inferior to adalimumab + MTX in terms of DAS28-CRP in RA patients with an inadequate response to MTX [6]. In addition, the FINCH 3 trial showed that filgotinib 200 mg/day monotherapy was higher than MTX in the proportion of achieving clinical remission in active RA patients with limited or no prior exposure to MTX [31]. However, to date, no study has performed a head-to-head comparison of JAK inhibitors versus IL-6 inhibitors for patients with RA. The JAK inhibitors demonstrate effectiveness by directly inhibiting the JAK-STAT signaling pathway, whereas the IL-6 inhibitors, such as tocilizumab, also indirectly inhibit the JAK-STAT pathway by inhibition of IL-6 signaling [4]. Therefore, the effectiveness of both drugs, which demonstrate a similar mechanism of action, should be compared for patients with active RA.

The strength of this study is its design as a randomized prospective evaluation of therapeutic efficacy using not only clinical disease activity indices but also MSUS, which accurately and objectively evaluates disease activity at the joint level in a patient series drawn from multiple centers with a standardized evaluation by MSUS. The study also provides a comprehensive analysis of the serum levels of many biomarkers, such as cytokines and chemokines. Because each treatment has a different method of administration, we were not able to use a blinding approach for treatment selection. However, the evaluators of the clinical disease activity indices and the evaluators of MSUS will be separated and blinded to each evaluation. This study directly compares the effectiveness of filgotinib and tocilizumab, which has not been examined to date. Finally, we will be able to evaluate the effectiveness of both drugs using MSUS and serum biomarkers, as well as clinical disease activity indices, patient-reported outcomes, and radiographic images from various angles.

## Trial status

The TRANSFORM study received ethical approval on February 12, 2021. Recruitment started in April 2021 and is expected to finish by September 30, 2024.

## Supplementary Information

**Additional file 1.** SPIRIT 2013 Checklist.

**Additional file 2.** Informed Consent Form.

## Data Availability

The datasets used or analyzed (or both) during the current study are available from the corresponding author on reasonable request.

## Abbreviations

ACR: American College of Rheumatology
bDMARD: Biological disease-modifying antirheumatic drug
CDAI: Clinical disease activity index
CRP: C-reactive protein
csDMARD: Conventional synthetic disease-modifying antirheumatic drug
DAS28: Disease Activity Score-28
DMARD: Disease-modifying antirheumatic drug
EDC: Electronic data capture
EQ-5D-5L: EuroQol 5 Dimensions 5-Level
ESR: Erythrocyte sedimentation rate
EULAR: European League Against Rheumatism
FACIT-F: Functional Assessment of Chronic Illness-Fatigue
GS: Grayscale
HAQ-DI: Health Assessment Questionnaire Disability Index
IFN: Interferon-gamma
IL: Interleukin
JAK: Janus kinase
JCR: Japan College of Rheumatology
MMP-3: Matrix metalloproteinase-3
MSUS: Musculoskeletal ultrasound
MTX: Methotrexate
OMERACT: Outcome Measures in Rheumatology
PD: Power Doppler
RA: Rheumatoid arthritis
SDAI: Simplified disease activity index
SMO: Site management organization
STAT: Signal transducer and activator of transcription
TNF: Tumor necrosis factor
vdH-mTSS: Van der Heijde-modified total Sharp score
