# Supplementary material for: Efficacy and safety of selective JAK 1 inhibitor filgotinib in active rheumatoid arthritis patients with inadequate response to methotrexate: comparative study with filgotinib and tocilizumab examined by clinical index as well as musculoskeletal ultrasound assessment (TRANSFORM study): study protocol for a randomized, open-label, parallel-group, multicenter, and non-inferiority clinical trial
Source: Trials. 2023 Mar 3;24:161. doi: 10.1186/s13063-023-07176-5 (PMC9985291; doi:10.1186/s13063-023-07176-5)
Supplement: Supplementary file 2 — Additional file 2. Informed Consent Form. [file 13063_2023_7176_MOESM2_ESM.docx]

**For Patients**

**Efficacy and safety of selective JAK 1 inhibitor Filgotinib in active rheumatoid arthritis patients with inadequate response to methotrexate: Comparative study with Filgotinib and Tocilizumab examined by clinical index as well as musculoskeletal ultrasound assessment**

**(TRANSFORM STUDY)**

**Creation Date: October 27, 2021**

**Ver. 1.4**

**(Name of medical institution) (Name of department)**

**Introduction.**

　This booklet describes the " Efficacy and safety of selective JAK 1 inhibitor Filgotinib in active rheumatoid arthritis patients with inadequate response to methotrexate: Comparative study with Filgotinib and Tocilizumab examined by clinical index as well as musculoskeletal ultrasound assessment

(TRANSFORM STUDY)" which is being conducted at multiple centers and led by the Department of Immunology and Rheumatology, Nagasaki University Hospital.

**1. clinical research**

　The diagnosis and treatment of each disease has evolved over a long period of time. In addition, the advancement and development of medicine will continue to be important to provide more effective and safer treatment for patients. Such advances and developments in diagnostic and treatment methods require a great deal of research, some of which must be conducted on healthy people and patients. This is called clinical research. Clinical research is only possible with the understanding and cooperation of patients.

The clinical research we are about to describe has also been reviewed and approved by the Nagasaki University Clinical Research Review Committee, which is accredited by the Minister of Health, Labor and Welfare. After obtaining the approval of the administrator of the conducting medical institution, the implementation plan for conducting clinical research is submitted to the Minister of Health, Labor and Welfare.

**Participation in clinical research is voluntary.**

　Please listen to the explanation of this study, fully understand its contents, and make a free choice as to whether you wish to participate in this study. You will not be penalized in any way for refusing to participate. If you choose to participate, please sign the "Consent form" and give it to the investigator.

・Withdrawal of Consent to Participate in Research

Even if you have agreed to participate in this research, you may withdraw at any time. If you wish to do so, please give the investigator a signed withdrawal form or tell him or her orally.

If you decide to withdraw from the study, you will not be disadvantaged in your future treatment.

If you withdraw from the study, and still do not agree to the use of your specimens and information from this study after withdrawing your consent, all specimens and information will be destroyed.

**3. the diseases that are the subject of this study**

Rheumatoid arthritis is a disease that causes synovitis in multiple joints due to an unexplained immune abnormality, resulting in joint pain and swelling. An important aspect of the treatment of rheumatoid arthritis is not only to relieve joint pain and swelling, but also to reduce joint destruction caused by persistent inflammation. To this end, several anti-rheumatic drugs are currently being used.

Methotrexate, a conventional anti-rheumatic drug, is the first-line treatment for rheumatoid arthritis. However, a certain percentage of patients are methotrexate-resistant, meaning that they cannot be treated with methotrexate because methotrexate is ineffective or causes side effects when taken. Therefore, it is important to choose which drug to use next to treat methotrexate-resistant patients. In the past, biologics such as TNF inhibitors, interleukin-6 (IL-6) inhibitors such as tocilizumab, and T-cell co-stimulation modulators have been used primarily for methotrexate-refractory patients. JAK inhibitors are oral medications, unlike biologics, which are intravenous or subcutaneous injections. Clinical studies have shown that JAK inhibitors are no less effective than TNF inhibitors, although JAK inhibitors and TNF inhibitors act in different ways, and both JAK inhibitors and IL-6 inhibitors act in a similar way by reducing the action of IL-6, a protein that causes inflammation. However, JAK inhibitors and IL-6 inhibitors act on similar parts of the body, such as suppressing the action of IL-6, a protein that causes inflammation. Therefore, we believe that research comparing the efficacy of JAK inhibitors and IL-6 inhibitors is needed.

In the treatment of rheumatoid arthritis, advances have been made not only in treatment but also in imaging tests. In recent years, musculoskeletal ultrasound (MSUS) has become an increasingly common imaging test in daily practice. MSUS can visually detect inflammation of the synovial membrane in joints, which is central to the pathophysiology of rheumatoid arthritis, and can be used to investigate the activity of the disease. It is believed to provide a more acute picture of inflammation than the combined examination and blood tests that are often used as indicators of rheumatoid arthritis activity. In addition, MSUS is painless, harmless, and can be performed repeatedly.

However, the previous studies discussed above were evaluated using a measure of activity based on a combination of examination and blood tests, and did not use MSUS.

**4. purpose of the research**

The purpose of this study is to evaluate the efficacy of filgotinib (JAK inhibitor) or tocilizumab (IL-6 inhibitor) in patients with rheumatoid arthritis who are methotrexate-resistant (ineffective, side effects occur) by switching treatment to filgotinib (JAK inhibitor) or tocilizumab (IL-6 inhibitor), and to evaluate whether filgotinib is no less effective than tocilizumab, using examination findings and blood The objective is to evaluate the efficacy of filgotinib as noninferior to tocilizumab by using MSUS in addition to examination findings and blood tests. We will also examine in more detail how these two drugs benefit rheumatoid arthritis by evaluating in detail whether there were any differences in MSUS and blood test results between treatment with filgotinib and treatment with tocilizumab. These studies will help us provide better care for patients with symptoms similar to yours.

**5. the nature of the research**

(1) Eligible patients

　　Patients who meet the following conditions (criteria) are eligible

1. Patients aged 20 years or older at the time of providing consent.
2. Patients diagnosed as having RA according to the 2010 RA

classification criteria of the American College of Rheumatology/European League Against Rheumatology.

1. Patients with moderate or more disease activity (DAS28-ESR ≥ 3.2) at the time of the eligibility evaluation.
2. Patients who have received MTX treatment continuously for at least 8 weeks before the time of providing consent and in whom MTX at the same dose of 8 to 16 mg per week for at least 4 weeks was ineffective. However, administration of less than 8 mg will be included in cases in which there is intolerance to 8 mg or more per week.
3. Patients who can personally provide written consent at their own free will after being receiving a thorough explanation of the study and fully understanding their participation in the study.

However, patients who meet the following conditions (criteria) are not eligible to participate in the study.

1. Patients treated with more than 5 mg per day of prednisolone.
2. Patients in whom the items for the contraindication of filgotinib and tocilizumab apply.
3. Patients with a history of using JAK inhibitors and IL-6 inhibitors.
4. Patients in whom the dosage of csDMARDs and glucocorticoids, was modified within 4 weeks prior to the time of providing consent.
5. Patients who have received treatment with TNF inhibitors (ie, infliximab, infliximab BS, adalimumab, adalimumab BS, golimumab, and certolizumab pegol), and abatacept within 8 weeks prior to the time of providing consent.
6. Patients who have received TNF inhibitors (ie, etanercept, and etanercept BS) within 4 weeks prior to the time of providing consent.
7. Patients who have used prohibited concomitant drugs and prohibited concomitant treatments, other than csDMARDs and the biological preparations noted above, within 4 weeks prior to the time of providing consent.
8. Patients with concurrent illness causing musculoskeletal disorders other than RA (ie, ankylosing spondyloarthritis, reactive arthritis, psoriatic arthritis, crystal-induced arthritis, systemic lupus erythematosus, systemic scleroderma, inflammatory myopathy, and mixed connective tissue disease).
9. Women who are breastfeeding, pregnant, or wish to fall pregnant, and those who do not consent to use contraception from the time of the eligibility evaluation until 12 months after the final dose of the investigational drug.
10. Individuals who, for other reasons, are deemed ineligible of study participation by the principal investigator.

In addition to this, your treatment history, current medical condition, and medications you are taking will be reviewed by the investigator to make a comprehensive determination as to whether you are eligible to participate in the study.

In addition, even after you have participated in the study, if the research physician determines that you should not participate in this study, we will discontinue your participation. Please understand that we may have to discontinue your participation against your will. The investigator will continue to provide you with the best possible treatment after discontinuation.

(2) Research Methods

Patients with rheumatoid arthritis refractory to methotrexate will be switched from methotrexate to filgotinib (200 mg once daily, although 100 mg may be required depending on your renal function) or tocilizumab (162 mg once every two weeks by subcutaneous injection). The activity of rheumatoid arthritis will be evaluated using medical examinations, blood tests, and imaging tests such as MSUS.

Patients who participate in this study have a 1 in 2 chance of being assigned to one of two groups: the filgotinib group or the tocilizumab group. Neither you nor the study physicians will be able to choose which group you will be in. You will be assigned to one group or the other in a lottery-like fashion.

(3) Drugs/treatments to be used

Filgotinib, the drug used in this study, is a JAK inhibitor, a type of drug that suppresses the action of a protein called cytokine, which causes inflammation and joint destruction, by suppressing an enzyme in cells called "JAK. It is manufactured and marketed by Gilead Sciences, Inc., and is a new drug that has just been approved for the treatment of rheumatoid arthritis.

Tocilizumab is a type of IL-6 inhibitor, which suppresses the action of IL-6, one of the cytokines present in our immune system to cause inflammation. It is manufactured and marketed by Chugai Pharmaceutical, Inc., and is used worldwide as a treatment for rheumatoid arthritis.

(4) Schedule

The information to be collected in this study and the timing of the collection are listed in the table below.

| (data) item | | Pre-observation period | Research Observation Period | | | | | | time of discontinuance |
| --- | --- | --- | --- | --- | --- | --- | --- | --- | --- |
|  |  |  | At 0 week (baseline evaluation) | 2 week time | At 4 weeks | At 8 weeks | At 12/36 weeks | 24/52 weeks |  |
| tolerance level | | After obtaining consent to the time of baseline evaluation | nashi (Pyrus pyrifolia, esp. var. culta) | ±3 days | ±7 days | ±7 days | ±14 days | ±14 days | Within 14 days from the date of discontinuation |
| Obtaining Consent | | ● | - | - | - | - | - | - | - |
| Confirmation of patient background and eligibility | | ● | - | - | - | - | - | - | - |
| Research Drug Administration | | - |  |  |  |  |  |  | - |
| Confirmation of medication and dosage status | | - | ● | ● | ● | ● | ● | ● | ● |
| vital signs | | - | ● | ● | ● | ● | ● | ● | ● |
| Tender and swollen joints | | ● | ● | ● | ● | ● | ● | ● | ● |
| Physician VAS and Patient VAS | | ● | ● | ● | ● | ● | ● | ● | ● |
| DAS28-ESR | | ● | ● | ● | ● | ● | ● | ● | ● |
| DAS28-CRP | | - | ● | ● | ● | ● | ● | ● | ● |
| CDAI/SDAI | | - | ● | ● | ● | ● | ● | ● | ● |
| ACR20 /ACR50 /ACR70 | | - | - | ● | ● | ● | ● | ● | ● |
| HAQ-DI | | - | ● | ● | ● | ● | ● | ● | ● |
| EQ-5D-5L | | - | ● | ● | ● | ● | ● | ● | ● |
| FACIT-F | | - | ● | ● | ● | ● | ● | ● | ● |
| Duration of morning stiffness  and activity assessment | | - | ● | ● | ● | ● | ● | ● | ● |
| blood test | | ● | ● | ● | ● | ● | ● | ● | ● |
| image | MSUS | - | ● | - | ● | - | ● | ● | ● |
|  | Radiography | - | ● | - | - | - | - | ● | - |

- The pre-trial visit and the 0-week visit (baseline evaluation) can be performed on the same day.
   If they are performed on different days, the results of the Week 0 (baseline evaluation) visit can be substituted for the results of the Week 0 visit.

(5) Investigation items, observation items, and inspection items

Participants will undergo the following tests to assess changes since the beginning of the study.

| (data) item | Contents and Evaluation Methods |
| --- | --- |
| Patient Background | Date of birth, gender, height, weight, comorbidities, date of diagnosis of rheumatoid arthritis, previous positive and highest rheumatoid factor (RF) and anti-citrullinated peptide antibody (ACPA) levels, date methotrexate started, current methotrexate dosage, history of biologic therapy, smoking history, previous therapy |
| Confirmation of medication and dosage status | Check medication and dosing compliance with filgotinib or tocilizumab. |
| vital signs | Body temperature, blood pressure, pulse rate |
| Tender and swollen joints | Sixty-eight tender joints and 66 swollen joints are evaluated. |
| Physician assessment of general disease activity Visual analogue scale (VAS) | This is an evaluation to be completed by the physician. |
| Patient assessment of general disease activity and pain Visual analogue scale (VAS) | This is an evaluation to be completed by the patient. |
| DAS28-ESR,.  DAS28-CRP | It is a measure of the intensity of rheumatoid arthritis activity. |
| CDAI/SDAI | It is a measure of the intensity of rheumatoid arthritis activity. |
| ACR20 /ACR50 /ACR70 | The degree of improvement of rheumatoid arthritis is evaluated by combining several evaluation indices. |
| Health Assessment Questionnaire-Disability Index (HAQ-DI) | This is a questionnaire evaluation of physical function to be completed by the patient. |
| EQ-5D-5L | This is a questionnaire evaluation of health status (quality of life) to be completed by the patient. |
| FACIT-F | This is a questionnaire evaluation of health status (fatigue) to be completed by the patient. |
| Duration of morning stiffness  and activity assessment | Using the morning stiffness evaluation sheet, the time that the morning stiffness lasted  and assess the degree of movement and pain. |
| blood test | The examination items vary depending on the time of visit.  <Inspection items  Hematological examination: white blood cells, white blood cell classification (neutrophil count, lymphocyte count, monocyte count, eosinophil count, basophil count), red blood cells, hemoglobin, platelets, hemopancreas  Blood biochemical tests: sodium, potassium, chlor, urea nitrogen, creatinine, CK, total bilirubin, AST, ALT, ALP, LDH, gamma-GTP, CRP  RF, ACPA, MMP-3: Indicators of immune abnormalities in rheumatoid arthritis.  Cytokines and other biomarkers: These are substances in the blood that are mainly related to inflammation and immunity. The following items are measured  EGF, Eotaxin, FGF-2, Flt-3L, Fractalkine, G-CSF, GM-CSF, GRO, IFNα2, IFNγ, IL-1α, IL-1β, IL-1ra, IL-2, IL-4, IL-5, IL-6, IL-7, IL-8, IL-10, IL-12 (p40 ), IL-12 (p70), IL-13, IL-15, IL-17A, IL-17F, IL-18, IL-22, IL-27, IP-10, MCP-1, MCP-3, MDC, MIP-1α, MIP-1β, sCD40L, TGFα, TNFα, TNFβ, VEGF, PDGF-AA, VCAM-1, ICAM-1 (measured by multi-suspension array), IL-6 (measured by ELISA), TNFα (measured by ELISA) |
| MSUS | 22 joints (interphalangeal [IP] joints, proximal interphalangeal [PIP] joints, metacarpophalangeal [MCP] joints, and wrist joints) are evaluated. |
| Radiography | Take radiographs of the frontal surfaces of both hands and both toe joints. |

(6) Participation period

The period of participation in this study is 13 months from the date of consent.

(7) Treatment after completion of the study

After the study is completed, we will provide appropriate treatment according to your medical condition and status. If you have any questions, please contact the investigator at any time.

**6. other treatment methods**

If you choose not to participate in this study, the investigator will still provide you with the best possible care for your medical condition.

**Planned duration of the study and number of participants**

(1) Research implementation period

This study will run from the date of publication in jRCT until September 30, 2024.

(Application period: until September 30, 2022)

- jRCT: Database maintained by the Ministry of Health, Labour and Welfare (see Chapter 11)

(2) Number of expected participants

The study will involve a total of 400 patients at all participating sites.

**8. expected benefits and disadvantages**

**(side effects/complications)**

(1) Projected profit

By participating in this study, you will be closely examined and tested, which will allow us to assess disease activity in more detail than usual and provide a more complete picture of your rheumatoid arthritis condition. In addition, the results of the tests performed in this study may help us to better understand your health status. By participating in this study, you can reduce your treatment burden.

Furthermore, the results of this research may contribute to future medical advances.

(2) Anticipated disadvantages

If you participate in this study, you may have the following disadvantages The physicians in charge of this study will always check your health status and will try to minimize the burden on you.

1. The following events may occur as described in "(3) Predicted Side Effects/Complications" below.
2. We will draw approximately 20 mL more blood per visit than normal. This amount is medically considered to have no effect on your disease or course of treatment.
3. Although this study will involve the use of radiation, the amount of radiation used in this study is almost the same as that used in a normal medical examination, and therefore, from a medical point of view, it is not considered to affect your health.
4. Compared to general practice treatment, the number of visits, hospital stays, and examinations may increase.

(3) Anticipated side effects/complications

Filgotinib and tocilizumab, the medications used in this study, have been reported in previous studies to cause the following side effects

filgotinib

1. Side effects that can have a significant impact on your health
2. **infectious disease**

**Infections (including opportunistic infections) such as shingles (0.2%) and pneumonia (0.3%) may occur.**

1. **Perforation of the gastrointestinal tract** (frequency unknown)
2. Neutropenia (0.1%), lymphopenia (<0.1%), hemoglobin decreased (anemia: frequency unknown)
3. **impairment of liver function**

Hepatic dysfunction such as increased ALT (0.8%) and AST (0.7%) may occur.

1. Interstitial pneumonia (frequency unknown)
2. Venous thromboembolism (frequency unknown)

Pulmonary embolism and deep vein thrombosis may occur.

1. Other side effects

|  | 1% or more but less than 10 | 0.1% or more but less than 1 |
| --- | --- | --- |
| Infectious and parasitic diseases | Urinary tract infection, upper respiratory tract infection |  |
| Blood and lymphatic system disorders |  | neutropenia |
| Nervous System Disorders | floating dizziness |  |
| gastrointestinal disorder | nausea |  |
| clinical examination |  | Increased creatine phosphokinase in blood |

Tocilizumab

- 1. Side effects that can have a significant impact on your health

**1) Anaphylactic shock (frequency unknown), anaphylaxis (0.3%)**

**After injection, hypotension, dyspnea, loss of consciousness, dizziness, nausea, vomiting, scratchiness, and flushing may occur.**

**2) Infectious diseases**

**Severe infections such as pneumonia (3.6%), herpes zoster (2.8%), infectious gastroenteritis (2.3%), cellulitis (2.1%), infectious arthritis (0.2%), sepsis (0.3%), nontuberculosis antimycobacteria (0.3%), tuberculosis (frequency unknown), pneumocystis pneumonia (frequency unknown) may occur.**

**3) Interstitial pneumonia** (frequency unknown)

Interstitial pneumonia may occur.

4) Intestinal perforation (0.2%)

Intestinal perforation may occur.

**5) Blood disorders**

Agranulocytosis (frequency unknown), leukopenia (7.3%), neutropenia (6.4%), and thrombocytopenia (1.8%) may occur.

6) Heart failure (frequency unknown)

Heart failure may occur.

1. Hepatic dysfunction (frequency unknown)

Significant liver dysfunction with elevations in AST, ALT, bilirubin, etc. may occur.

1. Other side effects

|  | 1% or more | Less than 1 | frequency unknown |
| --- | --- | --- | --- |
| resistance mechanism failure | Herpesvirus infection, influenza | Oral candidiasis, parotitis | wound infection |
| respiratory impairment | Upper respiratory tract infection (nasopharyngitis, upper respiratory tract infection, etc.), sinusitis, bronchitis, sore throat, cough, rhinitis | Asthma, pleurisy, rhinorrhea | Pharyngeal discomfort, hemoptysis, pharyngeal erythema, epistaxis, bronchiectasis, nasal obstruction |
| metabolic disorder | Increased cholesterol, increased LDL, increased triglycerides, hyperlipidemia, increased HDL, hypercholesterolemia | LDH elevated, CK elevated, blood uric acid increased, diabetes exacerbated, serum ferritin decreased, blood phosphorus decreased | Hypertriglyceridemia, decreased total protein, decreased blood potassium, increased blood glucose, increased blood phosphorus, decreased blood calcium |
| liver damage | ALT increased, γ-GTP increased, AST increased, liver function abnormal, bilirubin increased, ALP increased | Fatty liver, cholelithiasis |  |
| circulatory disturbance | Hypertension, elevated blood pressure | Supraventricular extrasystoles, ventricular extrasystoles, partial ST depression, partial ST elevation, hypotension, palpitations | T-wave inversion, T-wave amplitude decrease, T-wave amplitude increase |
| Blood and coagulation disorders | Anemia, increased eosinophil count, decreased fibrinogen, decreased lymphocyte count | Increased neutrophil count, increased white blood cell count, lymphadenitis | Increased fibrin degradation products (FDP, D-dimer), decreased hemoglobin, lymphadenopathy, decreased hematocrit, decreased red blood cell count, increased TAT |
| gastrointestinal disorder | Gastroenteritis, stomatitis, diarrhea, abdominal pain, constipation, cheilitis, vomiting, reflux esophagitis, gastric and intestinal polyps, periodontal disease, dental caries | Hemorrhoids, abdominal discomfort, nausea, abdominal distention, gastric ulcer, indigestion, anorexia, glossitis | Acute pancreatitis, dry mouth, toothache |
| psychoneurotic disorder | Headache, floating dizziness, insomnia | Sensory loss, peripheral neuropathy |  |
| ear damage | Otitis media, vertigo | Otitis externa, tinnitus, sudden hearing loss | ear ache |
| eye disorder | conjunctivitis | Conjunctival hemorrhage, ocular dryness, ophthalmoplegia, chalazion, blepharitis, vitreous floaters, retinal hemorrhage | cataract (opacity in lens of eye) |
| skin disorder | Rash [eczema, prurigo, papules, etc.], pruritus, nail infection, skin infection, urticaria, erythema, ringworm, keratosis, alopecia | Subcutaneous bleeding, dry skin, ingrown toenails, blisters, skin ulcers | Dermoid cysts, acne |
| Musculoskeletal disorders | backache | Arthralgia, myalgia (myalgia, stiff shoulders), osteoporosis, neck pain, decreased bone density | Limb pain, juvenile arthritis exacerbation |
| urinary disorders | Cystitis, positive urinary red blood cells, urinary tract infection | Urine protein, pyelonephritis, urinary sugar, frequent urination | Increased BUN, kidney stones, increased NAG, positive urinary leukocytes |
| genital disorder | vaginal infection | genital bleeding | cervical polyp |
| Other | Injection site reactions [erythema, itching, swelling, bleeding, hematoma, pain, etc.], weight gain, fever, allergic rhinitis, abscess, edema | Seasonal allergies, chest pain, fatigue, dyspepsia, moodiness, chest discomfort, hot flashes, chills | Immunoglobulin G decreased, thrombophlebitis, positive rheumatoid factor, positive DNA antibody, positive antinuclear antibody, flushing, increased CRP |

Other unknown side effects may also occur.

　During the study period, we will carefully monitor you for any side effects or other symptoms that may not be good for you. If you experience any unusual symptoms, please inform the investigator immediately.

**9. please observe the following**

Please observe the following while participating in the study

1. Please follow the instructions of the investigator during the study period.
2. Please come to the clinic on the assigned day. In the unlikely event that you are unable to come to the hospital due to an inconvenience, please contact us in advance.
3. If you see another doctor or visit another healthcare provider during the study period, please inform them that you are participating in this study. Also, please notify your investigator of any additions or changes in your medications.
4. Please be sure to use contraception during the study period. In the unlikely event that you become pregnant, please inform your research physician immediately.
5. Please inform your investigator immediately if you have any unusual symptoms.

**10. if the research is discontinued**

Even if you participate in this study, we will discontinue your participation in the following cases, taking into consideration the ethical and safety implications of your participation.

Please understand that we may have to discontinue the study against your will. After discontinuation, the research physician will continue to provide you with the best possible treatment.

1. Filgotinib withdrawal for more than 7 consecutive days in the filgotinib-treated group
2. In the tocilizumab-treated group, tocilizumab was withdrawn for more than 2 consecutive
3. If you wish to discontinue your participation in this study
4. If you find that you do not meet the study's entry requirements
5. If you are unable to come to the hospital on a certain day or otherwise unable to participate in this study
6. If you find out you are pregnant
7. If this entire study is terminated
8. If the physician in charge of the research determines that the research should be terminated due to your condition of illness or course of treatment
9. Other cases in which the investigator deems it advisable to discontinue the study

**11. information about the research**

During the course of the study, we will promptly inform you of any new information that may affect your safety or willingness to participate in the study. You are free to decide whether or not you wish to continue participating in clinical research.

This study will disclose items that the World Health Organization requires to be disclosed in conducting research and other items that contribute to ensuring transparency in the process of clinical research and the public's choice to participate in clinical research by registering them in a database maintained by the Ministry of Health, Labour and Welfare (hereinafter referred to as "jRCT". jRCT = Japan Registry of Clinical Trials URL: ). The Ministry of Health, Labour and Welfare (MHLW) will disclose such matters by registering them in the jRCT database (jRCT = Japan Registry of Clinical Trials URL: https://jrct.niph.go.jp/). Personal information in this study will be protected so that it will not be known.

In addition, the research protocol and materials on research methods related to the conduct of this research may be obtained or viewed, if you so request, to the extent that it does not interfere with the protection of the personal information of other research subjects or with the securing of such clinical research.

**12. provision of test results**

If any of the tests performed for this study are directly related to your medical care, the results will be explained to you by the investigator in the same manner as for normal medical care. However, the results of MSUS examinations will not be provided during the course of this study.

Other test results that are not directly related to medical treatment will not be provided, but will be explained to you if you wish, so please ask your investigator.

**13. protection of personal information**

　To protect personal information, a number (identification number) is assigned to each patient, and this number is used when handling specimens and information, and no personally identifiable information is used. A correspondence list will be created to link you to this identification number. This correspondence list will be kept in the hospital and will not be taken out of the hospital.

In addition, in order to ensure that this research is being conducted properly, the people involved in the study (Nagasaki University Hospital research staff, companies contracted by Nagasaki University Hospital, the Nagasaki University Clinical Research Review Committee, and Ministry of Health, Labor and Welfare officials) may have direct access to your medical records, but they will do so based on confidentiality obligations and the Personal Information Protection Law. Therefore, there is no need to worry about your privacy-related information (address, name, phone number, etc.) being leaked to outside parties. In addition, you will not be identified in any reports or other documents.

The results of this study may be published at conferences or in medical journals. In such cases, your personal information will not be disclosed.

Please note that by signing the consent form, you are also agreeing to the above, so please include this point if you agree.

**14. handling of specimens and information obtained in this study**

(1) Handling of specimens and information

A portion of the blood samples you provide for this study will be sent to SRL Corporation to measure the components of your blood. In addition, image data from MSUS and joint X-ray will be sent to Nagasaki University Hospital for evaluation. As described in "13. Protection of Personal Information," the specimens will be provided with an identification number assigned to them so that personal information will not be leaked to outside parties.

In addition, some of your information obtained for this study will be registered in the EDC system, EDC stands for Electronic Data Capture, which is a system used to collect information necessary for the study via the Internet. The registered information will be managed under strict security and only some limited staff members involved in this study will have access to it, so your personal information will not be leaked to outside parties.

(2) Storage of specimens and information

Information obtained from this research will be kept at each medical institution for five years after the completion of the research. Of the specimens sent to SRL Corporation, those remaining after testing will also be sent to Nagasaki University Hospital, and will be stored within Nagasaki University Hospital for 5 years after the completion of the research. These specimens and information will be kept as long as possible after 5 years from the end of the research.

When disposing of specimens and information, we will follow the rules of each medical institution and take sufficient care not to leak personal information outside the institution.

(3) Use of specimens and information for other research

The specimens and information obtained from this study may be used in other studies for further examination of this study and for clarification of rheumatoid arthritis disease. In such cases, there is a possibility that your specimens and information will be provided to other institutions. Even in this case, your personal information will not be leaked to outside parties. If your specimens and information are to be used in a new study, it will be conducted after review and approval by the Ethics Review Committee in the same manner as this study. At this time, we do not plan to share anonymized information with anyone other than the medical institutions conducting this study.

If you do not wish your specimen or information to be used or provided in another study, please notify your investigator. This will not be detrimental to your future treatment.

(4) Disclosure of inspection results, etc.

If you would like to see your personal information yourself, please consult your investigator. Any disclosure of your personal information will always be made to you by the principal investigator and the research physician. However, we will not necessarily disclose all individual information to you, since there is a concern that information that is unclear about the significance of the results obtained may be misleading to you or your relatives. In addition, information that cannot be used to identify you will not be disclosed to you as personal information.

**15. response and compensation in the event of a health hazard**

If you experience any adverse health effects as a result of your participation in this study, the investigator will take appropriate measures and provide adequate medical treatment.

Since this research is to be conducted within the scope of normal insurance treatment, health insurance will be used to cover medical expenses for treatment in the event of health problems. In principle, you will be responsible for paying the co-payment of medical expenses.

However, compensation will be provided for health hazards that are judged to have a clear causal relationship to this research through the clinical research insurance that this research is covered by. Please ask your research physician about the details of the compensation and the conditions under which it is covered. Please note, however, that you may not be eligible for compensation if you do not follow the instructions of the investigator or if your health damage is deemed to have been caused intentionally or through gross negligence.

If you wish to receive compensation, or if you would like to discuss whether or not you are eligible for compensation, please contact your doctor or the contact person in charge of your case. In order to receive compensation, you will be required to provide your name, address, health damage situation, bank account number, and identification documents, which will not be used for any purpose other than compensation payment.

**16. cost sharing**

(1) Costs to be borne

In this study, you will be treated with either filgotinib or tocilizumab. The cost of each medication will vary, but you will pay your co-pay for the medication and tests using your health insurance, just as you would pay for regular medical care.

However, you will not be responsible for the cost of MSUS and some blood tests (RF, ACPA, MMP-3, cytokines, and other biomarkers) performed for this study. Gilead Sciences, Inc., the fund provider for this study, will cover the costs.

(2) Payment of burden reduction expenses

Participation in this study will require more visits and more tests than usual treatment. Therefore, while you are participating in the study, we will provide you with 4,000 yen in the form of a Quo card for each visit at 0, 2, 4, 8, 12, 24, 36, and 52 weeks and at the time of discontinuation as a burden reduction fee from the research fund. However, if the visit at the time of discontinuation is on the same day as the above visits, no Quo card will be provided at the time of discontinuation.

**17. conflict of interest and funding sources for research**

　A conflict of interest is a situation in which a third party may be concerned that the research is not being conducted fairly and appropriately, such as falsification of research data or preferential treatment of certain companies due to financial interests with outside parties.

　This research is funded by Gilead Sciences, Inc. and uses drugs manufactured and marketed by Chugai Pharmaceutical Co. The physician in charge of conducting this research has prepared a Conflict of Interest Management Standard and a Conflict of Interest Management Plan regarding the status of conflicts of interest in this research, and has submitted them to the Nagasaki University Clinical Research Review Committee. The research is being conducted only after it has been judged that there are no ethical problems and that the research can be conducted in a fair manner. Although some of the principal investigators and subinvestigators in this research have conflicts of interest with companies involved in the research, we will manage them appropriately in accordance with the Clinical Research Act and related notifications.

**18. about monitoring**

　In order to ensure that your confidentiality is maintained and that the study is being conducted properly, the person assigned by the principal investigator to review the data may have access to your medical records and other data related to the study at the hospital. Again, such persons are required by law to maintain confidentiality and will do so in a manner that protects your privacy.

**19. ownership of intellectual property rights**

　Results from this research may yield intellectual property, such as patent rights, in which case the intellectual property rights do not belong to the patient.

**20. the structure for conducting this research**

This study will be conducted at multiple centers throughout Japan, with the Department of Rheumatology and Collagen Diseases, Nagasaki University Hospital, as the lead institution.

≪Principal Investigator.

Name: Atsushi Kawakami

Dept: Department of Rheumatology and Collagen Disease, Nagasaki University Hospital

Address: 1-7-1 Sakamoto, Nagasaki City

Phone: 095-819-7260

≪Principal investigator at ●● Hospital.

Name: ●●●●

Department: ●●●●

Address: ●●●●

Phone: ●●●●

**21. contact information and contact person**

If you have any questions, concerns or questions about this study, please do not hesitate to contact the research physician.

≪doctor in charge of research.

- - 1. research physician

Name: ●●●●

Department: ●●●●

Phone: ●●●●

**22. consultation desk for comments and complaints (excluding those related to the content of medical treatment and clinical research) (*Can be deleted in accordance with the operation of each medical institution)**

A consultation service is available for patients and their families.

Phone: ●●●●

Reception hours: ●●●●

**23. regulations surrounding this research**

To ensure your human rights and safety, this research will be conducted in accordance with the following laws and regulations

- Declaration of Helsinki (wherein Hirohito renounced the Helsinki Declaration)^※^
- Clinical Research Act (Act No. 16 of 2017) and related notices (effective April 2018)

*: Refers to the "Recommendations for Physicians Engaged in Biomedical Research Involving Human Subjects" adopted by the General Assembly of the World Medical Association in 1964.

For Hospital Preservation

ver. 1.4

Consent form

(Name of medical institution)

(Affiliation) (Principal investigator or administrator of the institution)

Title of the study:

Efficacy and safety of selective JAK 1 inhibitor Filgotinib in active rheumatoid

arthritis patients with inadequate response to methotrexate: Comparative

study with Filgotinib and Tocilizumab examined by clinical index as well as

musculoskeletal ultrasound assessment (TRANSFORM STUDY)

[Explanatory notes

| 1. clinical research | 13. protection of personal information |
| --- | --- |
| 2. participation in clinical research | 14. handling of specimens and information obtained in research |
| 3. about your disease | 15. response and compensation in the event of a health hazard |
| 4. purpose of the research | 16. cost sharing |
| 5. the nature of the research | 17. conflict of interest and funding sources for research |
| 6. other treatment methods | 18. about monitoring |
| Planned duration of the study and number of participants | 19. ownership of intellectual property rights |
| 8. anticipated benefits and disadvantages | 20. implementation structure |
| 9. please observe the following | 21. contact information |
| 10. if the research is discontinued | 22. consultation desk for comments and complaints |
| 11. information about the research | 23. regulations surrounding this study |
| 12. provision of test results |  |

I have explained this study to the patient.

Explanation Date: Year Month Day

Name of person(s) providing explanation: (Signature) 　　　　　　　　　　　　　(Self-signed)

[Patient's signature line].

I have received an explanation of the above-mentioned research, understand its contents, and agree to participate in this research of my own volition. I will receive a copy of the explanation and this consent document.

Date of Consent: Year Month Day

Patient's name: (Self-signed)

I assisted the research physician in explaining the study to the patient.

Explanation Date: Year Month Day

Name of person(s) assisting the explanation: (Signature) 　　　　　 　　　　　　(Signature)

For Patients

ver. 1.4

Consent form

(Name of medical institution)

(Affiliation) (Principal investigator or administrator of the institution)

Title of the study:

Efficacy and safety of selective JAK 1 inhibitor Filgotinib in active rheumatoid

arthritis patients with inadequate response to methotrexate: Comparative

study with Filgotinib and Tocilizumab examined by clinical index as well as

musculoskeletal ultrasound assessment (TRANSFORM STUDY)

[Explanatory notes

| 1. clinical research | 13. protection of personal information |
| --- | --- |
| 2. participation in clinical research | 14. handling of specimens and information obtained in research |
| 3. about your disease | 15. response and compensation in the event of a health hazard |
| 4. purpose of the research | 16. cost sharing |
| 5. the nature of the research | 17. conflict of interest and funding sources for research |
| 6. other treatment methods | 18. about monitoring |
| Planned duration of the study and number of participants | 19. ownership of intellectual property rights |
| 8. anticipated benefits and disadvantages | 20. implementation structure |
| 9. please observe the following | 21. contact information |
| 10. if the research is discontinued | 22. consultation desk for comments and complaints |
| 11. information about the research | 23. regulations surrounding this study |
| 12. provision of test results |  |

I have explained this study to the patient.

Explanation Date: Year Month Day

Name of person providing explanation: (Signature)

[Patient's signature line].

I have received an explanation of the above-mentioned research, understand its contents, and agree to participate in this research of my own volition. I will receive a copy of the explanation and this consent document.

Date of Consent: Year Month Day

Patient's name: (Self-signed)

I assisted the research physician in explaining the study to the patient.

Explanation Date: Year Month Day

Name of person(s) providing assistance : (Signature)

For Hospital Preservation

ver. 1.4

Consent Withdrawal Letter

(Name of medical institution)

(Affiliation) (Principal investigator or administrator of the institution)

Title of the study:

Efficacy and safety of selective JAK 1 inhibitor Filgotinib in active rheumatoid

arthritis patients with inadequate response to methotrexate: Comparative

study with Filgotinib and Tocilizumab examined by clinical index as well as

musculoskeletal ultrasound assessment (TRANSFORM STUDY)

[Patient's signature line].

　I hereby withdraw my consent to participate in the 　above-mentioned research, which I have received from the physician incharge of the research and have given my consent to participate in this research.

- - Please do not use any inspection or other data obtained.
  - There is no problem in using the data obtained from inspections, etc.

(↑Please check one of the boxes □)

Date of withdrawal of consent: Month/Year

Patient's name: (Self-signed)

Signature of the physician in charge of the research

I confirm that the above patient has withdrawn his consent.

Date of confirmation: Year Month Day

　　　　　Name of person confirming: (Signature)

For Patients

ver. 1.4

Consent Withdrawal Letter

(Name of medical institution)

(Affiliation) (Principal investigator or administrator of the institution)

Title of the study:

Efficacy and safety of selective JAK 1 inhibitor Filgotinib in active rheumatoid

arthritis patients with inadequate response to methotrexate: Comparative

study with Filgotinib and Tocilizumab examined by clinical index as well as

musculoskeletal ultrasound assessment (TRANSFORM STUDY)

[Patient's signature line].

　I hereby withdraw my consent to participate in the 　above-mentioned research, which I have received from the physician incharge of the research and have given my consent to participate in this research.

- - Please do not use any inspection or other data obtained.
  - There is no problem in using the data obtained from inspections, etc.

(↑Please check one of the boxes □)

Date of withdrawal of consent: Month/Year

Patient's name: (Self-signed)

Signature of the physician in charge of the research

I confirm that the above patient has withdrawn his consent.

Date of confirmation: Year Month Day

　　　　　Name of person confirming: (Signature)
